# Effects of mindfulness-based interventions on cognition in people with multiple sclerosis: a systematic review and meta-analysis of randomized controlled trials

**DOI:** 10.3389/fpsyt.2024.1339851

**Published:** 2024-07-12

**Authors:** Alyssa Komar, Kirsty Dickson, Mohammad Alavinia, Tania Bruno, Mark Bayley, Anthony Feinstein, Jillian Scandiffio, Robert Simpson

**Affiliations:** ^1^ Department of Medicine, Division of Physical Medicine and Rehabilitation, University of Toronto, Toronto, ON, Canada; ^2^ NHS Lothian, Edinburgh, Scotland, United Kingdom; ^3^ Department of Medicine, Division of Psychiatry, University of Toronto, Toronto, ON, Canada; ^4^ St. Michael’s Hospital, Toronto, ON, Canada; ^5^ University of Glasgow, Glasgow, Scotland, United Kingdom

**Keywords:** mindfulness, multiple sclerosis, cognitive function, systematic review, meta-analysis

## Abstract

**Introduction:**

Cognitive impairment affects up to 65% of people with multiple sclerosis (PwMS), undermining functional independence and quality of life. The objective of this study is to synthesize existing randomized controlled trial (RCT) evidence on the effects of Mindfulness-based interventions (MBIs) on cognitive function in PwMS.

**Methods:**

A systematic literature search was conducted to identify RCTs assessing MBIs effects on cognitive functioning in PwMS. Using pre-defined criteria, two independent reviewers screened titles, abstracts, and extracted data from included studies. Meta-analysis was performed, where possible, using a random effects model. Narrative synthesis was undertaken. Preferred Reporting Items for Systematic Reviews and Meta-analysis guidance was followed. PROSPERO_ID:(CRD42021286429).

**Results:**

Twelve eligible RCTs were identified, n=700 PwMS. MBIs included both standardized and tailored interventions, in-person and virtually. A variety of measures of cognitive functioning were reported. Five studies (n=254 PwMS) were included in meta-analysis; pooled results suggested MBIs effectively improved scores on the Paced Auditory Serial Addition Test (PASAT)-2 (SMD=0.38; 95% CI 0.06-0.71; I2 63%; p=0.02), whereas improvements were of borderline significance on the PASAT-3 (SMD=0.32; 95% CI -0.01-0.64; I2 65%; p=0.06), and, although trending to positive, were statistically insignificant on the Perceived Deficits Questionnaire (SMD=0.34; 95 CI -0.05-0.74; I2 0%; p=0.09) and Symbol Digits Modality Test (SMD=0.25; 95% CI -0.15-0.66; I2 0%; p=0.21).

**Conclusion:**

Preliminary findings in meta-analysis are inconsistent but suggest potential benefits from MBI training on cognitive functioning in PwMS. High quality RCTs are necessary to test more definitively the impact of MBIs on cognitive functioning in PwMS.

**Systematic review registration:**

PROSPERO, identifier CRD42021286429.

## 1 Introduction

Multiple sclerosis (MS) is a chronic, progressive, neurodegenerative condition (1) and the major cause of inflammatory neurologic disability in young adults (2, 3). MS can adversely impact multiple functional domains including visual, vestibular, sensory, motor, affective, and cognitive. Indeed, cognitive impairment is particularly prevalent among people with multiple sclerosis (PwMS) affecting 34% to 65%, and dysfunction correlates most robustly with increased age, longer disease duration, progressive MS phenotype, co-morbid depression, and fatigue (4). The most commonly impaired cognitive function in PwMS is information processing speed, with attention, working memory, long term memory, and executive function also commonly affected (5).

While the etiology of cognitive impairment in PwMS is not fully understood, inflammation and structural brain damage can result in functional disconnection/synaptic failure (6). This has been attributed in large part to white matter lesions, as demonstrated in a 2017 meta-analysis which confirmed a modest correlation between total brain white matter lesions and cognitive impairment in PwMS. More specifically, a correlation was found between white matter lesion burden and impaired cognition as measured by the Symbol Digits Modality Test (SDMT), a measure of information processing speed, and by the Paced Auditory Serial Addition Test (PASAT), a measure of working memory, divided attention and information processing speed (7). However, cognitive impairment in PwMS is likely multifactorial and related to both white and grey matter damage (6). Grey matter lesions and atrophy appear to have an important role, generally (8), whilst, more specifically, thalamic and hippocampal volume correlate with memory impairment, and basal ganglia with attentional impairment (8–10). Functional brain imaging studies using magnetic resonance imaging (fMRI) have demonstrated altered cerebral activation patterns in PwMS both at rest and during tasks that target attention, memory, and information processing speed (11–15). Such functional reorganization may serve as a compensatory and adaptive response to structural brain damage and facilitate cognitive functioning, but it is also associated with increased cognitive dysfunction (5). Indeed, it is thought that over time cumulative structural brain damage in PwMS leads to decreased network efficiency and eventual ‘categorical’ cognitive impairment (16).

Impaired cognitive functioning in PwMS is also linked to comorbidities, including cardiovascular, endocrine, and psychiatric (17), physical symptoms, such as fatigue, pain, and sleep dysfunction (18), affective symptoms such as stress, anxiety, and depression (19), commonly prescribed medications (such as antiepileptics (20), anticholinergics (21)), polypharmacy more generally (22), ‘self-medication’ strategies (such as cannabis use) (23), or lifestyle factors (such as smoking) (24). All of these represent modifiable risk factors either through preventative approaches or targeted treatment.

The World Health Organization stipulates that rehabilitation ‘addresses the impact of a health condition on a person’s everyday life by optimizing their functioning and reducing their experience of disability’ (25). Fundamentally, rehabilitation is based on a biopsychosocial model of illness. Cognitive rehabilitation for PwMS can be viewed as seeking to minimize the disabling effects of impairments, by means that promote direct recovery or adaptation of body functions, increased independence in functional activities, and greater societal participation. This is achieved largely through treating or eliminating contributory factors such as comorbidities, symptoms, personal or environmental issues. Therefore, cognitive rehabilitation is by definition a complex intervention with multiple potential active and interacting components and is likely modified by context (26).

The current evidence base for cognitive rehabilitation for PwMS is limited. A recent systematic review of 87 studies found insufficient evidence to recommend any pharmacological agents. Individual studies of symptomatic treatments have demonstrated mixed results, whilst studies of disease modifying treatments (DMTs) have frequently not included cognitive outcomes (27). In terms of behavioural interventions, a Cochrane Review of neuropsychological rehabilitation for PwMS in 2014 found low-level evidence for cognitive training in improving attention and memory in PwMS (28), whilst a 2016 Cochrane Review found memory rehabilitation can be effective for improving verbal memory and information processing speed, as well as QoL in PwMS. The latter review criticized the quality of existing evidence and the ecological validity of outcome measures used in clinical trials (29). Another systematic review of cognitive rehabilitation for PwMS, including 33 studies but only 7 RCTs, assessing a wider range of rehabilitative strategies, reported considerable heterogeneity in terms of treatment modalities, cognitive domains targeted, and treatment outcomes reported. The authors indicated supportive evidence for the majority of interventions but delivered an overall assessment rating of ‘inconclusive’ (30).

Mindfulness-based interventions (MBIs) are increasingly used to help people manage long-term disabling conditions. Deriving from Buddhist and Yogic meditation techniques, MBIs teach group participants to become mindful through meditations focused on breath, body, and movement, in addition to psychoeducation on stress, reflective group discussion, and regular home practice (31, 32). Mindfulness has been defined as “paying attention in a particular way: on purpose, in the present moment, and non-judgmentally” (33), hinting at a key role for fundamental aspects of cognitive processing. Although the mechanisms of action are incompletely understood, theoretical models suggest instrumental roles for attentional training and emotional regulation (34). In meta-analyses, mediating factors include improvements in mindfulness (35), cognitive and emotional reactivity (36), executive skills, such as meta-awareness (37), and the amount of home practice completed (38). MBIs are themselves complex interventions, and ‘common factors’ such as instructor characteristics, group processes, and peer support also contribute to effects observed following treatment (39).

MBIs are also associated with functional and structural neuroplastic effects. A recent systematic review identified enhanced amygdala-frontoparietal functional connectivity on fMRI following mindfulness training, thought to reflect improved emotional regulation. In addition, increased connectivity between attention and salience networks was linked with improved awareness (40). MBIs are also linked to improvements in many symptoms which are common among PwMS, including stress, anxiety, depression, and fatigue (41, 42), factors well known to moderate cognitive functioning. For example, anxiety and depression worsen memory, information processing speed, and executive function in PwMS (19). Taken together, there is a need to establish the effects of MBIs on cognitive functioning in PwMS and, to our best knowledge, no previous evidence synthesis has systematically explored this question.

The objective of this systematic review and meta-analysis is to explore the evidence for the effectiveness of MBIs in improving cognitive function in PwMS.

## 2 Methods

### 2.1 Protocol and registration

A protocol was registered prospectively with PROSPERO, Centre for Reviews and Dissemination, University of York: CRD42021286429.

### 2.2 Inclusion criteria

Eligible studies were identified based on SPIO criteria – Study design, Population, Intervention, and Outcome (43). To be eligible for inclusion, studies had to be RCTs comparing an MBI to an active comparator or care as usual. Participants had to be PwMS of any age and phenotype. The intervention(s) being tested had to include core practices of Mindfulness-based stress reduction (MBSR) and/or Mindfulness-based cognitive therapy (MBCT), namely mindful breathing, mindful body awareness, and mindful movement. Only validated outcome measures (subjective or objective) of cognitive functioning were considered.

### 2.3 Search strategy

We employed a comprehensive search strategy for use in six major electronic databases, including the Allied and Complementary Medicines Database (AMED), Cumulative Index of Nursing and Allied Health Literature (CINAHL), Cochrane Central Register of Controlled Trials, ExcerptaMedicadataBASE (EMBASE), Medical Literature Analysis and Retrieval System Online (MEDLINE), and PsycINFO. The initial search was in April 2021, and was updated in May 2023. We searched from 1980 to the date of search, given that MBIs were first developed and piloted in the 1980s. We included only studies published in English, among human subjects, in the peer-reviewed academic literature.

### 2.4 Study selection, storage, and screening

Search results were first imported into COVIDENCE, a systematic review data storage software package. Three independent reviewers (AK, KD, JS) screened study titles/abstracts for potential eligibility using the keywords ‘mindfulness’ and ‘multiple sclerosis’. The same three independent reviewers further assessed selected studies against SPIO criteria to determine definitive eligibility. A senior party reviewer adjudicated any disagreements (RS).

### 2.5 Data collection/data items

Data from the final list of included studies were extracted by three independent reviewers (AK, KD, JS), guided by the Preferred Reporting Items for Systematic Reviews and Meta-Analyses (PRISMA), with intervention programming documented using the Template for Intervention Description and Replication (TIDieR) (44).

### 2.6 Quality appraisal

The Cochrane Collaboration’s tool for assessing the risk of bias (RoB) was used to summarize risk for individual outcomes in selected studies, graded as high, unclear, or low risk (45). This assessed generation of sequence, concealment of allocation, blinding of participants, outcome assessors and personnel, incomplete outcomes, selective reporting of outcomes, and any other source of bias. Finally, an overall RoB within each trial was determined based on the number of individual outcomes falling into the high, unclear, and low risk categories:

Low = Low RoB for all key domains.

Unclear = Low or unclear RoB for all key domains.

High = High RoB for one or more key domains.

### 2.7 Meta-analysis

Four separate meta-analyses were conducted to determine the overall mean difference between mindfulness-based interventions (MBIs) and cognitive function in PwMS, which is defined as PASAT-2, PASAT-3, Perceived Deficits Questionnaire (PDQ) and SDMT. The heterogeneity among the studies was evaluated using the chi-square test and the I² statistic, which quantifies the proportion of variation in the effect estimates attributable to heterogeneity rather than random chance. When the heterogeneity test showed statistical significance (I² > 50% and p < 0.05), a random effects model was used; otherwise, a fixed effects model was employed. The meta-analyses were performed with the ReviewManager (RevMan) software (Version 5.4.1, Nordic Cochrane Centre, Cochrane Collaboration, 2011), with statistical significance set at p < 0.05. Effect sizes and standard mean differences were calculated using RevMan software.

### 2.8 Primary summary measures

The main objective for this study was to determine the impact of MBI on cognitive functioning. Main outcome measures were all reported as continuous with mean and standard deviation (SD) values, plus the number of participants for each treatment group extracted.

### 2.9 Synthesis of results

Throughout the study, we adhered to the PRISMA guidance (46).

## 3 Results

**Figure 1:**
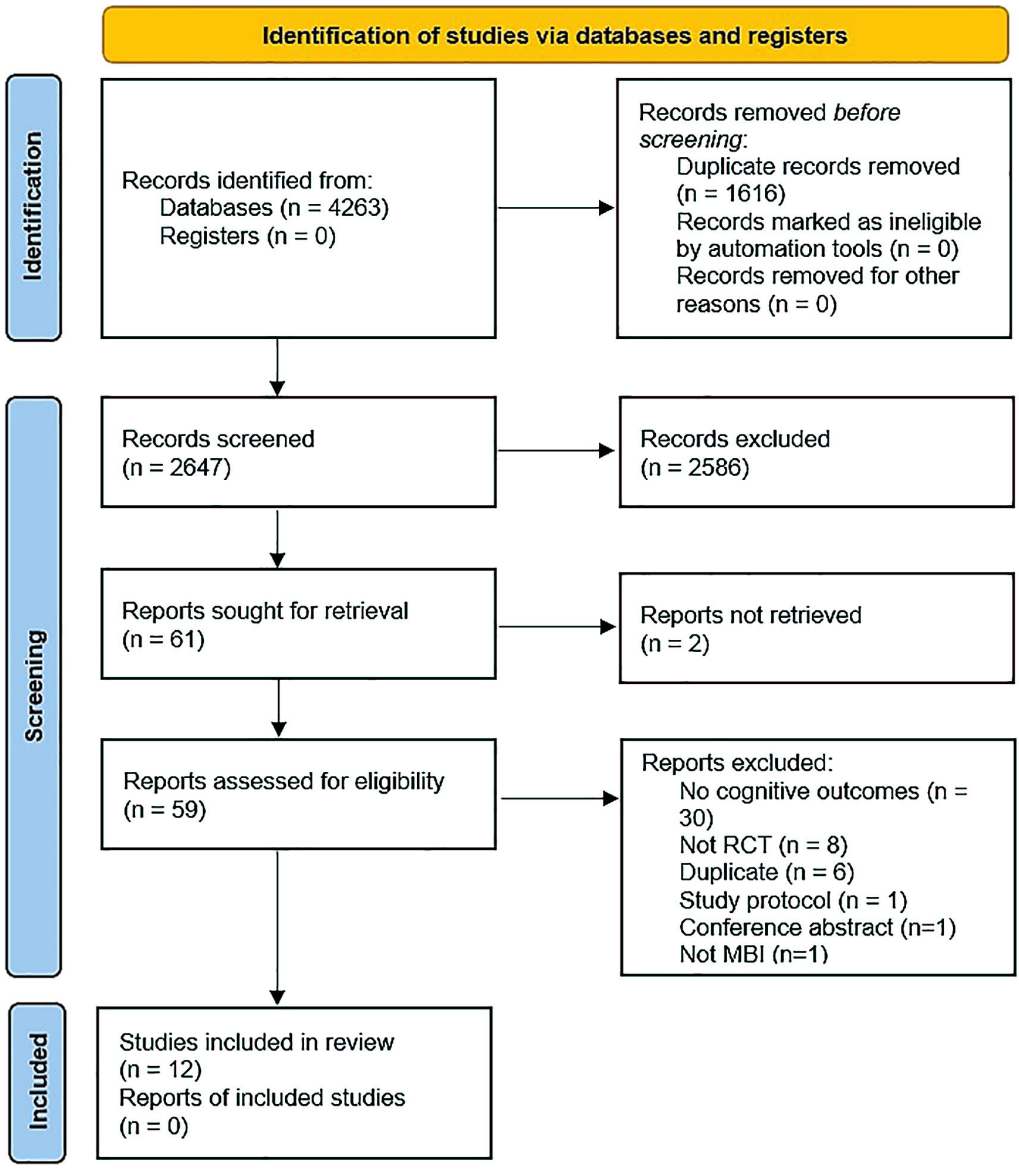
PRISMA flow diagram.

We identified 12 RCTs as eligible for inclusion in the systematic review, with five studies reporting endpoint data on the same outcomes that were usable in meta-analysis (**Figure 1**). Where relevant, we sought additional information from study authors; however, none replied.

### 3.1 Systematic review

#### 3.1.1 Study characteristics

One study (47) performed secondary analyses of a pilot RCT. Three studies took place in Iran (48–50), three in the USA (47, 51, 52), and one each in Switzerland (53), Scotland (54), Spain (55), Canada (56), the Netherlands (57), and Germany (58). Five studies tested an MBI against treatment as usual (50, 53–56), one compared against both treatment as usual and cognitive therapy (57), and four studies compared to an active comparator, including psychoeducation (52) and cognitive training (47, 51, 58). Two studies did not specify control conditions (48, 49). Six studies were statistically powered (47, 51–53, 56, 57). The number of study participants ranged from 24-150 (median 60.5). One study reported measuring outcomes at five points in time (baseline, immediately post-intervention, 3-, 6- and 12-months later) (58), four studies reported measuring outcomes at three points in time (baseline, immediately post-MBI, follow up that varied from three months to one year post-MBI) (52, 54, 56, 57), while five studies measured pre-post measurements only (47–51), with one study measuring pre- and 6-months post (53) and one 12 months post (55) (**Table 1**).

**Table 1:** Study characteristics.

Study	Country	Study Design	Powered	Comparator	Sample size (n)	Study attrition	Cognitive outcome measure(s)	Data collection
Grossman et al. (53)	Switzerland	RCT	Yes	Treatment as usual	150	7%	• Neuropsychology assessment: Multiple Sclerosis Inventory of Cognition (MUSIC)	• Baseline • 6 months post MBI
Amiri et al. (48)*	Iran	RCT	No	Unclear	40	0%	• Wisconsin Card Sorting Test	• Baseline • Post MBI
Mahdavi et al. (49)	Iran	RCT	No	Unclear	24	NR	• Meta-Worry Questionnaire • Thought Fusion Inventory	• Baseline • Post MBI
Simpson et al. (54)	Scotland	RCT	No	Treatment as usual	50	12%	• Perceived Deficits Questionnaire • Emotional Liability Questionnaire	• Baseline • Post MBI • 3 months post MBI
Senders et al. (52)	USA	RCT	Yes	Psychoeducation	62	18%	• Paced Auditory Serial Attention Task 3	• Baseline • Post MBI • 12 months post MBI
De la Torre et al. (55)*	Spain	RCT	No	No mindfulness training, usual pharmacologic treatment	60	0%	• Wechsler Memory Scale-III • Symbol Digits Modalities Test (SDMT) • Controlled Oral Word Association Test (COWAT) • Paced Auditory Serial Addition Test 2, 3	• Baseline • 12 months post MBI
Schirda et al. (51)	USA	RCT	Yes	• Active cognitive training • Wait list control group	61	18%	• Difficulties in Emotion Regulation Scale • Ruminative Responses Scale & Penn State Worry Questionnaire composite score • Worry and Rumination Task	• Baseline • Post-MBI
Manglani et al. (47)	USA	RCT	Yes	• Active cognitive training • Wait list control group	61	18%	• Brief repeatable Battery of Neuropsychological Tests: • Word List Generation • 10/36 Spatial Recall Test • Selective Reminding Test • Symbol Digit Modalities Test • Paced Auditory Serial Addition Test 2,3	• Baseline • Post-MBI
Morrow et al. (56)	Canada	RCT	Yes	Treatment as usual	25	24%	• Multiple Sclerosis Neuropsychological Questionnaire (MSNQ)	• Baseline • Post-MBI • 6 months post-MBI
Nazaribadie et al. (50)*	Iran	RCT	No	Treatment as usual	53	12%	• Wisconsin Card Sorting Test • Paced Auditory Serial Addition Test 2, 3	• Baseline • Post-MBI
Baetge et al. (58)*	Germany	RCT	No	Metacognitive training, no mindfulness exercises	65	23%	• Perceived Deficits Questionnaire • Brief International Cognitive Assessment for MS (BICAMS) • Symbol Digit Modalities Test • Verbal Learning and Memory Test • Brief Visuospatial Memory Test revised (BVMT-R) • Weschler-Memory Scale • Multiple-Choice Vocabulary Intelligence Test	• Baseline • Post-MBI • 3 months post-MBI • 6 months post-MBI • 12 months post-MBI
Nauta et al. (57)*	Netherlands	RCT	Yes	• Cognitive rehabilitation therapy • Enhanced treatment as usual	110	14%	• Cognitive Failures Questionnaire • Behavior Rating Inventory of Executive Function-Adult Version (BRIEF-A) • Goal Attainment Scaling • Minimal Assessment of Cognitive Function in MS (MACFIMS) • Symbol Digit Modalities Test • Stroop Color-Word Test • California Verbal Learning Test • Brief Visuospatial Memory Test revised (BVMT-R) • Benton Judgment of Line Orientation Test • Controlled Oral Word Association Test • Delis-Kaplan Executive Function System Sorting Test	• Baseline • Post-MBI • 6 months post-MBI

*Study included in a meta-analysis.

MBI, mindfulness based intervention; NR, not reported; RCT, randomized control trial; USA, United States of America.

#### 3.1.2 Participant characteristics

Across the 12 RCTs, the total number of participants was 700, with 254 participants used to conduct the meta-analyses. Participant ethnicity was described in four studies (47, 51, 52, 54), most were Caucasian. Overall, the majority of participants were female (74%, n = 517), where reported (one study did not provide demographic characteristics of participants who discontinued the study (58)). The extractable mean participant age varied between 31.4 - 55.2 years [not reported in one study (49)]. Two studies reported on socioeconomic status (SES) (48, 54) and three studies provided information on participants’ employment status (54, 57, 58). All 12 studies provided information on education status, most having at least a high school education (47–57). Where reported, most (at least 515; 74%) had relapsing-remitting MS, at least 113 (22%) had secondary progressive MS, and at least 22 (3%) had primary progressive MS. Mean Expanded Disability Status Scale (EDSS) was reported in six studies with a range of 3.0-4.6 (47, 51–54, 58) and median EDSS was reported in two studies with a range of 2.0-4.0 (56, 57). Two studies reported on active comorbid conditions (54, 57) with five studies reporting on use of disease modifying drugs and/or psychotropic medications (52–55, 57). An interview was compulsory prior to taking part in three studies (49, 53, 55). One study required evidence of impaired mental wellbeing (stress, anxiety) at baseline in order to take part (52), one required participants with cognitive complaints (57), and one required impaired executive function (50) (**Table 2**).

**Table 2:** Participant characteristics.

Demographic	Grossman et al. (53)	Mahdavi et al. (49)	Simpson et al. (54)	Senders et al. (52)	Amiri et al. (48)	De la Torre et al. (55)	Schirda et al. (51)	Manglani et al. (47)	Morrow et al. (56)	Nazaribadie et al. (50)	Baetge et al. (58)	Nauta et al. (57)
Ethnicity, n (%)	NR	NR	White British 25 (100%)	White 60 (97%) Other 2 (3%)	NR	NR	White 44 (72%) Black 14 (23%) Biracial 2 (3%) Other 1 (2%)	White 44 (72%) Black 14 (23%) Biracial 2 (3%) Other 1 (2%)	NR	NR	NR	NR
Number of participants, n (%F)	150 (80%) 120	24 (100%) 24	50 (92%) 46	62 (78%) 48	40 (47.5%) 19	60 (67%) 40	61 (77%) 47	61 (77%) 47	25 (81%) 17	53 (60%) 32	65 (65%) 42	110 (75%) 82
Age, mean (SD)	47.3 (10.3)	NR	45 (10.9)	52.94 (11.37)	25.2 (4.5)	IG: 44.30 (10.34) CG: 48.80 (8.76)	45.7 (8.10)	45.7 (8.10)	37.1 (9.4)	IG 33.48 (8.59) CG 31.42 (6.58)	IG: 55.17 (6.61) CG: 51.85 (6.60)	48.7 (9.8)
SES	NR	NR	Postcode derived, controlled in analyses	NR	“Average or above average”	NR	NR	NR	NR	NR	NR	NR
Employed, n (%)	NR	NR	20 (40%)	NR	NR	NR	NR	NR	NR	NR	18 (28%)	39 (35%)
Education status	Mean 14.1 (SD: 1.9) years	Completed high school	56% university	60% at least college education	All high school diploma or university education	IG mean 1.77 (SD: 0.82) CG mean 1.53 (SD: 0.73)	Mean 16.0 years (SD: 2.27)	Mean 16.0 (SD: 2.27) years	Mean 14.5 (SD: 1.6) years	IG: 13.37 (SD: 2.33) CG: 13.62 (SD: 2.38)	30 had ‘high’ education (60%)	64 had ‘high’ education (58%)
Disease phenotype, n (%)	RR 123 (83%) SP 27 (18%)	NR	RR 40 (80%) SP 16 (32%) PP 4 (8%)	RR 41 (67%) SP 15 (25%) PP 4 (6%) Unknown 2 (3%)	NR	RR 60 (100%)	RR 59 (97%) PP 1 (1.5%) Unknown 1 (1.5%)	RR 59 (97%) PP 1 (1.5%) Unknown 1 (1.5%)	RR 25 (100%)	RR 30 (57%) SP 23 (43%)	RR 12 (24%) SPMS 38 (76%)	RR 66 (60%) SP 17 (15%) PP 12 (11%) Unknown 5 (5%)
EDSS score	Mean 3.0 (SD: 1.1)	NR	Mean 4.4 (SD: 1.8)	Mean 4.6 (SD: 1.93)	Range 0 – 5.5	NR	Mean 4.24 (SD: 1.31)	Mean 4.24 (SD: 1.31)	Median 2.0 (0.0-4.0)	IG 2.92 (SD: 0.74) CG 2.00 (SD: 0.63)	CG: 3.82 (SD: 1.4) IG: 4.48 (SD: 1.53)	Median 4.0 (2.0-8.0)
Comorbidity	NR	NR	Mean 2.4 (2.0); Range 0-9	NR	NR	NR	NR	NR	NR	NR	NR	CIRS median: 3 (3-9)
DMD use, n (%)	91 (60.1%)	NR	26 (52%)	34 (55%)	NR	NR	NR	NR	14 (67%)	NR	NR	58 (53%)
Psychotropic medication(s)	30 (20%)	NR	23 (46%)	35 (56%)	NR	NR	NR	NR	NR	NR	NR	NR

CG, control group; CIRS, Cumulative Illness Rating Scale; DMD, disease modifying drug; EDSS, Expanded Disability Status Scale; F, female; IG, intervention group; NR, not reported; PP, primary progressive; RR, relapse remitting; SD, standard deviation; SES, socioeconomic status; SP, secondary progressive.

#### 3.1.3 Intervention characteristics

Seven studies were based on MBSR (47, 51–55, 58), three on MBCT (48, 49, 57), one on the Mindfulness Ambassador Program (MAP) (56), and one on Metacognitive Model of Detached Mindfulness (50). Three studies reported on participant materials (47, 51, 54). All 12 studies reported on MBI session content, with two studies providing general details (53, 56). Nine studies described home practices (47, 51–58), whilst one study described this more generally (50). Eight studies reported on teacher characteristics (47, 50–54, 56, 57), but one study provided minimal detail (50). All 12 studies delivered group MBIs. Six studies reported intervention delivery location (47, 50, 51, 54, 57, 58), with two studies using a hybrid model of delivery (57, 58). One study had 10 weekly sessions (56), two studies had nine (53, 57), six studies had eight (48–50, 52, 54, 55), one study had seven sessions (58), and two studies had four weekly sessions (47, 51). Session length ranged from 1 to 2.5 hours, with one study (57) noting one session (i.e. silent retreat) that lasted 5 hours. Group class sizes ranged from 2 to 25 participants, with one or two instructors present. Seven studies tailored the MBI for PwMS (47, 51, 53–57), typically in advance, with two studies modifying movement exercises to accommodate physical impairments (54, 57). Home practice completion and session attendance were used to determine treatment adherence in nine studies (47, 48, 51–54, 56–58). The day retreat, characteristically part of week six in MBSR, was included in three studies (52, 53, 57).

#### 3.1.4 Outcome characteristics

All 12 studies assessed an aspect of cognitive functioning. Objective measures included the PASAT- 2 (47, 50, 55), PASAT- 3 (47, 50, 52, 55), SDMT (47, 55, 57, 58), Brief Visuospatial Memory Test revised (BVMT-R) (57, 58), Wisconsin Card Sorting Test (48, 50), Wechsler Memory Scale (55, 58), Controlled Oral Word Association Test (COWAT) (55, 57), Word List Generation (47), 10/36 Spatial Recall Test (47), Selective Reminding Test (47), Minimal Assessment of Cognitive Function in MS (MACFIMS) (57), Stroop Color-Word Test (57), California Verbal learning Test (CVLT) (57), Verbal Learning and Memory Test (58), Benton Judgement of Line Orientation Test (57), and the Delis-Kaplan Executive Function System sorting test (D-KEFS) (57). Subjective self-reported measures of cognitive functioning included the PDQ (54, 58), Behavior Rating Inventory of Executive Function-Adult Version (BRIEF-A) (57), Cognitive Failures Questionnaire (57) and MS Neuropsychological Questionnaire (MSNQ) (56). Other related assessments of cognitive functioning outcome measures included the, Emotional Liability Questionnaire (54), Difficulties in Emotion Regulation Scale (DERS) (51), Penn State Worry Questionnaire (PSWQ) composite score (51), Ruminative Responses Scale (51), Worry and Rumination task (51). Three studies completed comprehensive test batteries (53, 57, 58). Two studies reported mean daily home practice as 29.2 and 32.5 minutes (53, 54), two studies reported average total home practice of 817 minutes (47, 51), and one study reported median daily home practice as 38 minutes (52). Study attrition ranged from 0% to 26%.

### 3.2 Meta-analysis

**Figure 2:**
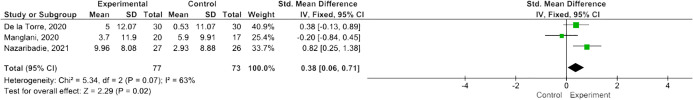
A forest plot showing the effects of MBI on the Paced Auditory Serial Addition Test-2.

**Figure 3:**
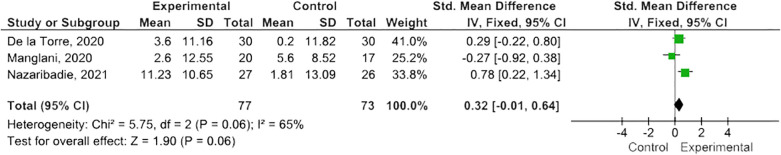
A forest plot showing the effects of MBI on the Paced Auditory Serial Addition Test-3.

**Figure 4:**
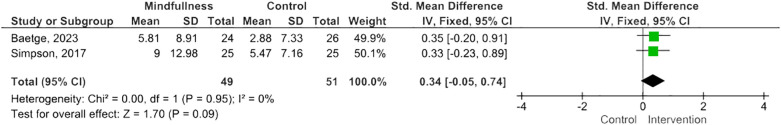
A forest plot showing the effects of MBI on the Perceived Deficits Questionnaire.

**Figure 5:**
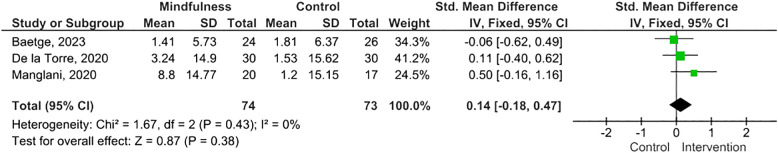
A forest plot showing the effects of MBI on Symbol Digit Modalities Test.

MBIs effectively improved scores on the PASAT-2 (SMD 0.38; 95% CI 0.06-0.71; p=0.02) though heterogeneity was moderate (I^2^ 63%) (**Figure 2**) (47, 50, 55). There was a trend towards improvement on the PASAT-3 with borderline significant results (SMD=0.32; 95% CI -0.01-0.64; I^2^ 65%; p=0.06) (**Figure 3**) (47, 50, 55). Benefits on the PDQ (SMD=0.34; 95 CI -0.05-0.74; I^2^ 0%; p=0.09) (**Figure 4**) (54, 58) and SDMT (SMD=0.14; 95% CI -0.18-0.47; I^2^ 0%; p=0.38) (**Figure 5**) (47, 55, 58) following MBI training were not statistically significant.

While two studies (48, 50) used the Wisconsin Card Sorting Test (WCST), one of these studies (48) did not report data that could be utilized in a meta-analysis. Additionally, two studies (57, 58) used the Brief Visuospatial Memory Test revised (BVMT-R), but one of these studies (57) did not report data that could be used in the meta-analysis. Another two studies (55, 57) used the Controlled Oral Word Association Test (COWAT), but one study (57) did not report data utilizable for the meta-analysis. One study reported data on information processing speed from the SDMT and Stroop Color-Word Test combined, and data could not be individually extracted for the SDMT (57).

### 3.3 Results by cognitive domain

#### 3.3.1 Subjective cognitive domains

In a feasibility RCT (n=50), subjective measure of cognitive function demonstrated significant improvement in attention post-MBI (p<0.05, d=0.62, CI 0.05-1.19) and prospective memory at 3 month follow up (p <0.05, d=0.81, CI 0.18-1.45), as assessed by the PDQ (54). There was no statistically significant difference post-MBI in overall cognition, retrospective memory, prospective memory, planning/organization, or at 3 month follow up in overall cognition, attention, retrospective memory, and planning/organization (54). Another study using the PDQ found MBI participants had improved retrospective memory, attention and concentration, and prospective memory both immediately post-MBI (p=0.006, d=0.62; p=0.01, d=0.55; p=0.002, d=0.73) and at 3 months follow up (p=0.02, d=0.61; p=0.03, d=0.53; p=0.02, d=0.62), though only improvements in prospective memory were significant at 6 months follow up (p=0.04, r = 0.52) (58). However, there was no significant benefit between those who took place in metacognitive training with mindfulness exercise compared to those who only participated in metacognitive training (F (1, 45) = 1.905, p = 0.174, partial η2 = 0.041, d = 0.41). In another study, the self-report MSNQ did not demonstrate statistically significant changes immediately post-MBI compared to the control group (p=0.066) or at 6 month follow up (p=0.896) in a pilot RCT (n=25) (56). Another study (57) found that MBCT had a positive effect on behavioral regulation as assessed by BRIEF-A at post-treatment compared to the enhanced treatment as usual (ETAU) group (β=− 3.6, p=.032, Cohen’s d=− 0.34); however, this was not sustained at 6-months follow-up. There was no significant difference in post-treatment subjective cognitive function as measured by the Cognitive Failures Questionnaire in the MBCT group compared to the ETAU group (β=− 4.8, p=.058, Cohen’s d=− 0.32) (57).

#### 3.3.2 Emotional regulation

No statistically significant difference in emotional lability was found post-MBI in a feasibility RCT, as assessed by the Emotional Lability Questionnaire (p=0.85, d=0.06, CI -0.42-0.51), nor at three months follow up (p=0.79, d=0.07, CI -0.39-0.30) (54). However, a pilot RCT (n=61) demonstrated statistically significant improvement in emotional dysregulation as assessed by Difficulties in Emotion Regulation Scale scores from pre- to post-training in the MBI group (p=0.01), and was significantly greater than the waitlist control group (p=0.002), however, effect sizes were not reported (51). There was also no statistically significant difference in the Worry and Rumination Task in emotion regulation strategies between MBI, active cognitive training, or waitlist groups over time (p=0.84) (51).

#### 3.3.3 Executive function

In one study, which used the Wisconsin Card Sorting Test (WCST), a measure of perseveration, set-shifting, and abstract thinking, there were no significant differences in the variables of categories number (p=0.65) or perseverative error (p=0.13) between MBI and control groups (n=40), but effect sizes were not reported (48). Similarly, another study found no significant differences in executive function, as measured by D-KEFS, between MBCT and control groups (p=0.59) (57). However, another study (50) found a statistically significant improvement post-MBI compared to the control group on the WCST variables of perseveration (n=53, p<0.01, d=0.48), total correct number (p<0.05, d=0.32), number of errors (p<0.05, d=0.39), first trial category (p<0.05, d=0.18); no statistically significant improvement between the intervention and control groups in the WCST variables of category (p>0.05, d=0.15), conception responses (p>0.05, d=0.18), other errors (p>0.05, d=0.20) (50). Perseverative cognition, as assessed by the Penn State Worry Questionnaire and Ruminative Responses Scale composite score, demonstrated statistically significant improvement post-MBI (p<0.001), as did change scores of the MBI group compared to the waitlist group (p=0.05) (51). Similarly, another study found that MBCT had a positive effect post-intervention on the Behavior Rating Inventory of Executive Function-Adult Version metacognition index (p=0.02); however, these findings were no longer significant at 6-months follow-up and effect sizes were not reported (57). In a quasi-experimental RCT (n=24), a statistically significant difference was found post-MBI in questionnaires that assessed metacognition, including the Meta-Worry Questionnaire (p=0.001) and Thought Fusion Inventory (p=0.006), however, effect sizes were not reported (49).

#### 3.3.4 Memory

In a pilot RCT (n=61), Wechsler Memory Scale-III components of Wechsler Long Term Memory (WLT) (p<0.001, d=0.516) and Wechsler Attention (WATT) (p<0.001, d=0.359), demonstrated statistically significant improvement post-MBI, with the control group also demonstrating a significant improvement in WATT (p<0.001). There was no statistically significant improvement post-MBI in Wechsler Short Term Memory (WST) (p=0.06), Wechsler Recognition (WREC) (p=0.35), or Wechsler Learning (WLEARN) (p=0.80), however, effect sizes were not reported (55). Selective Reminding Test (SRT) did not demonstrate statistically significant effects post-MBI on verbal learning and memory (p=0.61, n2p=0.020), and no statistically significant effect post-MBI on visuospatial learning and memory as assessed by the 10/36 Spatial Recall Test (p=0.18, n2p=0.065) (47). Another study used the Wechsler-Memory Scale to assess both verbal working memory and visuospatial working memory (58). Significant improvements in visuospatial working memory were seen post-MBI (p=0.03, d=0.59), but there were not significant differences between groups (p=0.27) (58). There were also no significant differences in verbal working memory between groups (p=0.86) (58). Another study used the California Verbal Learning Test and Brief Visuospatial Memory Test revised to assess immediate recall, long-term recall, and long-term recognition (57). No significant improvements were seen post-MBCT between groups.

#### 3.3.5 Verbal fluency

The Controlled Oral Word Association Test (COWAT) component of Verbal Fluency demonstrated a statistically significant difference post-MBI (p<0.001, d=0.305); however, there was no significant difference post-MBI in the COWAT component of Animals (p=0.07, ES not reported) in an RCT (n=60) (55). Another study reported no significant difference in COWAT post-MBCT (p=0.49), however, effect sizes were not reported (57). No statistically significant changes post-MBI were found from the Word List Generation test (p=0.43, n2p=0.032) (47).

#### 3.3.6 Comprehensive test batteries

Grossman et al. (53) described the Neuropsychological assessment, Multiple Sclerosis Inventory of Cognition (MUSIC), was administered pre-intervention and at 6-month follow up to assess short-term verbal memory, delayed recall, attention, information processing speed, verbal fluency, and cognitive interference and inhibitory control among 150 participants; however, follow up data was not reported (53). Baetge et al. (58) administered the Brief International Cognitive Assessment for Multiple Sclerosis (BICAMS) at baseline and follow-ups to examine information processing speed, verbal memory, and visuospatial memory, using the SDMT, Verbal Learning and Memory Test, and Brief Visuospatial Memory Test revised, respectively (58). There was no significant change in information processing speed nor verbal memory from baseline to follow-up nor between groups (p=0.59, d = 0.16; p=0.75, d = 0.06). There was significant worsening in visuospatial learning post-MBI, however this was seen in both non-MBI (p=0.046) and MBI groups (p=0.006). Contrarily, one study found a positive overall effect on processing speed post-MBI (β=0.2, p=.026, d = 0.20) and at 6-months follow-up (β=0.2, p=.027, d= 0.22) compared to the enhanced treatment as usual group (57).

### 3.4 Study quality

Study quality was highly variable. The assessment was frequently made challenging by incomplete reporting. Eight studies outlined random sequence generation (47, 50–54, 56, 57). Six studies were adjudged low risk for allocation concealment, with the remainder unclear (47, 51–54, 56). Blinding of assessors was outlined in seven studies (47, 51–54, 56, 57), with one study being assessed as high risk (50). Blinding of outcome assessment was outlined in six studies (47, 51–54, 56). Six studies were deemed low risk when assessing reporting of outcomes as incomplete (48, 50, 52, 54, 55, 58), and one study was high risk (47). Two studies were assessed as at high risk for selective reporting of outcomes (48, 53). In terms of overall within trials RoB assessments, two studies were deemed low risk (52, 54), two unclear (55, 56), and eight high risk (47–51, 53, 57, 58).

### 3.5 Adverse events

Two studies reported adverse events associated with MBI exposure (52, 54). In one study that used MBSR, a participant reported an episode of increased spasticity during mindful body awareness (52). In the same study another participant described increased anxiety following the MBSR day retreat (52). In another study using MBSR, one participant reported increased severity of chronic neuropathic pain following the ‘raisin exercise’ (54).

## 4 Discussion

### 4.1 Summary of main findings

Twelve RCTs that assessed the effects of an MBI on cognitive functioning in PwMS were eligible for inclusion in our systematic review. Out of these studies, eight reported cognition as the primary endpoint (47–51, 55, 57, 58), with four studies reporting cognition as a secondary measure (52–54, 56). From the 12 included studies, five had data extractable for use in our meta-analyses. In the meta-analyses, significant and borderline significant improvements were noted in the PASAT-2 and PASAT-3, respectively. Although trending to positive, no significant improvements were found on the PDQ or SDMT. The pooled result for SDMT differed from the two studies that both reported a significant change in the SDMT scores after MBI, as this meta-analysis compared the mean change from baseline between the control and MBI groups whereas the two studies reported on a within-group comparison. In our narrative synthesis, additional beneficial effects were reported in individual studies, pertaining to a wide range of cognitive functions, both fundamental (attention, memory), and higher order (executive function), suggesting a potential role for MBIs in improving information processing speed, attention, cognitive flexibility, calculation, emotion regulation, and meta-cognition. However, in making sense of these preliminary findings, it is necessary to highlight some important limitations identified in reviewing the studies included in this review.

Firstly, only one study recruited PwMS with baseline evidence of cognitive impairment as an eligibility criterion (50). This raises the risk for a ‘ceiling’ effect, whereby significant improvement may not reasonably be expected if participants are cognitively ‘intact’ according to scale criteria. Secondly, subjective self-report measures of cognitive function are notorious for not demonstrating robust correlations with objective measures. In PwMS, having a lower level of education, progressive phenotype, greater physical disability, and comorbid mood impairment are all known to lead to subjective overestimations of cognitive deficits (28). Thirdly, very few studies used composite assessments of cognitive functioning – this limits interpretation beyond the scope of individual tests, and whilst individual screening tests can have important predictive values [i.e., SDMT predicts cognitive relapses, employment (59)], such findings taken out of context can have limited ecological validity (60), or, more importantly, ability to inform patient need. Although not as comprehensive as a standardized clinical assessment with a neuropsychologist or specialist occupational therapist, comprehensive batteries (MACFIMS, BICAMS) provide the basis for a neuropsychological assessment, overview of impairments that can be captured psychometrically, and can predict task performance in activities of daily living (59).

### 4.2 Comparison with existing literature

The above limitations notwithstanding, MBIs may have a role to play in cognitive rehabilitation for PwMS, who value increased awareness of cognitive impairments, simple strategies for addressing difficulties, and group formatting which facilitates the normalization of symptoms and instrumental peer support processes (61). However, before a recommendation can be made for MBIs as part of comprehensive cognitive rehabilitation programming, more high-quality research is necessary. Indeed, MBIs are arguably better suited to addressing affective impairments, where existing evidence is strong for stress, anxiety, and depression – all are frequently comorbid and known to exacerbate cognitive dysfunction in PwMS. Stress has complex, bidirectional relationships with cognition in PwMS, increasing subjective sense of cognitive impairment, whereas executive dysfunction can predict greater comorbid stress, increased reading span predicting less comorbid stress. Executive dysfunction also predicts comorbid anxiety and depression in PwMS, whilst lower scores for anxiety correlate with better nonverbal memory, and better scores for depression are associated with improvements in attention and information processing speed (62–64).

While there is no other previous synthesis that has systematically explored the impact of MBIs on cognitive function in PwMS, a 2022 scoping review on third wave cognitive behavioural therapies in PwMS reviewed the impact of MBSR, MBCT, Acceptance and Commitment Therapy (ACT), and Dialectical Behaviour Therapy (DBT) separately (65). They found that MBSR was the most commonly studied approach. In addition, a 2022 systematic review and meta-analysis (66) examined Mindfulness and Acceptance-Based Interventions (MABIs) on a range of outcomes, including cognition, in PwMS. The interventions included MBIs, ACT, and DBT. This study demonstrated a moderate effect on attention (SMD 0.49; 0.19-0.80) and a large effect on memory (SMD 1.12; 0.06-2.17) (66); however, only one study assessing DBT was included (67) and only three (47, 54, 55) of the 12 RCTs identified in our current review were included, likely reflecting the earlier search cut-off date in their study. By comparison, our meta-analysis indicates that the effectiveness of MBIs at improving cognitive functioning in PwMS is inconsistent at best and no MBI can be said to be optimal in this context.

In non-MS populations, MBIs have been found to improve cognitive functioning generally, with a small but significant pooled effect size (g=0.15; CI 0.05 - 0.24), and small but significant pooled effects on executive function (g=0.15; CI 0.02 – 0.27) and working memory (g=0.23; CI 0.11 – 0.36). The clinical significance of this small effect is unclear, as the clinical populations included were diverse; only 14% were described as ‘individuals with neurocognitive disorders’, but analyses pooled these participants with people with other ‘psychiatric’ and neurological’ disorders. Without overt reporting of clinical ‘case-ness’, we cannot be sure about the degree of cognitive impairment, interpret response (or ‘remission’) in relation to baseline cognitive function. Overall, MBIs outperformed usual care, but not active comparators. Outcomes were moderated most by population (clinical vs non-clinical), comparator intervention type (active vs usual care), session duration and frequency (68). The latter finding makes sense intuitively, in that ‘dose’ has been identified as a mediating factor in beneficial outcomes associated with MBI in other meta-analyses (38).

### 4.3 Strengths of this review

Guided by the PRISMA checklist (46), the TIDieR checklist (44), and the Cochrane Collaboration RoB tool (45), our multidisciplinary team of reviewers used robust search, appraisal, and analysis techniques for extracting and analyzing data in this systematic review and meta-analysis.

### 4.4 Limitations of this review

Although we assessed quality using a reference standard, the Cochrane Collaboration RoB tool, we did not estimate the strength of any recommendation for the use of MBIs in PwMS. Future studies could do so by applying the Grading of Recommendations Assessment, Development and Evaluation (GRADE) criteria (69).

The standard deviations reported in the meta-analysis are to be considered an estimate as they were calculated based on a formula used for independent measures; however, they are dependent measures. The standard deviation of the mean differences was calculated based on the reported standard deviation across different populations, when it should be paired standard deviation provided by the data calculation.

Meta-analyses of RCTs by design exclude other potentially relevant data, such as that deriving from observational or qualitative research. When considering intervention feasibility, such as acceptability, accessibility, and implementability, as well as perceived effects from the point of view of participants, these alternate study designs can provide important insights into how and why interventions succeed or fail in a given context (61), and how they might be optimized to best meet patient needs and preferences.

### 4.5 Strengths and limitations of the included studies

Two studies were adjudged low, two studies as unclear, and eight studies as high RoB. Although all MS phenotypes were represented, by far the most participants had relapsing-remitting MS (74.3%), very few had primary progressive (3.4%), some had secondary progressive (21.9%), some had non-specified MS (1.3%), and none had progressive relapsing. This is similar to findings from a large international cohort study (n=2599) (70), which found that ~73% of their study population had relapsing-remitting MS. Furthermore, the mean sample age was relatively low at 31.4-52.9 years, as, arguably, was disability according to EDSS, whilst ethnicity, SES, and comorbidity were poorly covered, limiting the generalizability of the findings. Only three compared an MBI against an active comparator condition. Observed effects were mostly small, with a wide range of confidence intervals.

Given the well documented high levels of cognitive comorbidity in PwMS, it is notable that our meta-analysis has only been able to quantify the effects of MBI training on three commonly used but limited measures of cognition in PwMS. Other cognitive assessments were utilized in individual studies, where beneficial effects were reported, but meta-analysis was not possible. Future studies could address this evidence gap by measuring the impact of MBI training on cognition using a variety of subjective and objective assessments.

### 4.6 Implications for research

More, well-designed, high quality RCTs are needed to assess more definitively the impact of MBIs on cognitive functioning in PwMS. Although RCT methodology normally specifies a single primary outcome, this may have limited value in practice and instead it might make the most sense to study a composite cognitive ‘outcome’ using the BICAMS or MACFIMS. In addition, or alternatively, it may be prudent to consider MBIs as a ‘preventative’ strategy, rather than remediative, along the lines of building cognitive reserve/enrichment (71). Furthermore, MBIs have been reported to have a positive impact on biological markers of inflammation and aging, including telomere length, which hints at a potential neuroprotective effect (72). To the best of our knowledge, MBI impact on inflammatory markers in PwMS remains untested.

### 4.7 Implications for clinical practice

Currently, MBIs cannot be recommended as a mode of cognitive rehabilitation for PwMS. However, they do effectively improve common confounders such as stress, anxiety, depression, and fatigue so should be considered for these common comorbidities that often exacerbate cognitive difficulties in this population.

## 5 Conclusions

The impact of MBIs on cognitive functioning in PwMS remains unclear. Preliminary findings in meta-analyses are inconsistent but suggest potential benefits on information processing speed, cognitive flexibility, and calculation ability. Further, high-quality RCTs are necessary to test more definitively the impact of MBIs on cognitive functioning in PwMS. Such RCTs should assess impact on cognitive function across domains, using validated measures such as the SDMT, BICAMS or MACFIMS.

## Data availability statement

The original contributions presented in the study are included in the article/supplementary material. Further inquiries can be directed to the corresponding author.

## Author contributions

AK: Conceptualization, Data curation, Methodology, Writing – original draft. KD: Conceptualization, Data curation, Writing – review & editing. MA: Formal Analysis, Writing – review & editing. TB: Conceptualization, Writing – review & editing. MB: Conceptualization, Writing – review & editing. AF: Conceptualization, Writing – review & editing. JS: Data curation, Writing – review & editing. RS: Conceptualization, Writing – review & editing.
